# Pulmonary Mycobacterium avium Complex With Adenocarcinoma of the Lung: A Case Report

**DOI:** 10.7759/cureus.66589

**Published:** 2024-08-10

**Authors:** Priyavardhan Mishra, Mohit Kondisetti, Anant Patil, Nikhil Sarangdhar

**Affiliations:** 1 Medicine, DY Patil Deemed to be University School of Medicine, Navi Mumbai, IND; 2 Pulmonary Medicine, DY Patil Deemed to be University School of Medicine, Navi Mumbai, IND; 3 Pharmacology, DY Patil Deemed to be University School of Medicine, Navi Mumbai, IND

**Keywords:** nontuberculous mycobacteria (ntm), nontuberculous mycobacterial infection, antimicrobial therapy, lung cancer, adenocarcinoma, mycobacterium avium complex

## Abstract

Nontuberculous mycobacteria are responsible for causing pulmonary as well as extrapulmonary diseases. These organisms are often multidrug resistant and management of these cases poses a therapeutic challenge. Lung cancer has been a prevalent challenge globally with a high mortality rate in affected individuals. Adenocarcinoma poses debilitating outcomes in most patients by inflicting a diagnostic and therapeutic challenge. The concomitant association of adenocarcinoma and *Mycobacterium avium* complex worsens the prognosis causing a challenge in managing such cases. We present a rare association between adenocarcinoma and pulmonary *Mycobacterium avium* complex complicating the traditional therapeutic regime. A different approach in the administration of therapy for this unique concomitant association between two debilitating diseases is outlined in the presented report.

## Introduction

Pulmonary diseases caused by nontuberculous mycobacteria (NTM) are increasingly prevalent globally [1]. NTM are mycobacterial species that do not belong to the *Mycobacterium tuberculosis* complex or *Mycobacterium leprae* [2]. NTM are responsible for pulmonary and extrapulmonary diseases consisting of *Mycobacterium avium* complex (MAC) as the most common species [2]. MAC consists of multiple species, which are *M. arosiense*, *M. bouchedurhonense*, *M. timonense*, *M. vulneris*, and *M. yongonense* [2]. Chronic obstructive pulmonary disease, bronchiectasis, chronic aspiration or recurrent pneumonia, dormant or active tuberculosis (TB), pneumoconiosis, and bronchogenic carcinoma are the predisposing factors in 54-77% of pulmonary MAC disease patients [2]. Lung carcinoma has been categorized histopathologically into small-cell lung cancer (SCLC) and non-small-cell lung cancer (NSCLC), with NSCLC prevalent over 80% [3]. NSCLC consists of squamous cell carcinoma, adenocarcinoma (ADC), and large cell carcinoma, with ADC accounting for 40-45% of cases [3].

To our knowledge, we have not found any cases with a concomitant association of MAC infection and ADC. In our case, initiation of traditional antimicrobial therapy was ineffective in improving the patient’s vitals due to concomitant ADC bringing novelty for the presentation of the case.

## Case presentation

A 63-year-old, non-smoker, immunocompetent Indian male of low socioeconomic status reported to our outpatient clinic with symptoms of grade 2 modified Medical Research Council (MMRC) dyspnea for four to five years, for which he was not under any medication. Dyspnea worsened with exertion and was not relieved by rest. It was not accompanied by orthopnea or paroxysmal nocturnal dyspnea. He also experienced left-sided chest pain radiating to the back for two weeks and a loss of 11 kg of weight over the past two months. The patient had no history of diabetes mellitus, asthma, or hypertension and had not come across contact with any patient suffering from TB. During examination, the patient was afebrile with a pulse rate of 92 beats per minute with normal volume, a respiratory rate of 18 breaths per minute, blood pressure of 110/80 mmHg, and oxygen saturation of 90% on room air. Baseline hematological and serological tests, liver function test, renal function test, and urine analysis were found to be within normal range.

**Figure 1:**
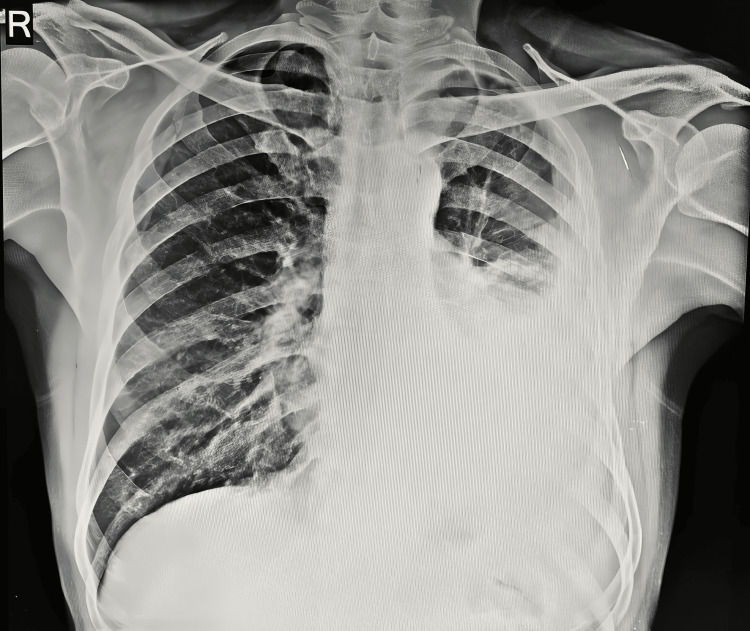
Chest X-ray showing left M2 + L2 homogeneous opacities with blunting of costophrenic angle.

**Figure 2:**
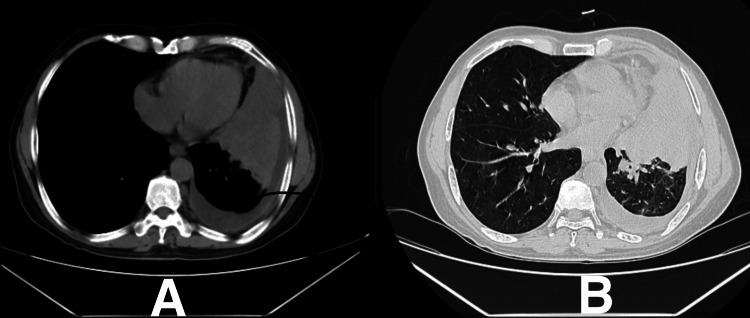
High-resolution computed tomography (HRCT) of the thorax showing volume loss due to a collapsed segment of the left lung, ipsilateral mediastinal shift due to central mass lesion, and centrilobular emphysematous changes over bilateral upper lobes. A: HRCT mediastinal window of the thorax. B: HRCT lung window of the thorax.

On chest X-ray (Figure 1), left M2 + L2 homogeneous opacities with blunting of costophrenic angle were observed. High-resolution computed tomography (HRCT) of the thorax (Figure 2) revealed mild volume loss due to a collapsed segment of the left lung, ipsilateral mediastinal shift due to central mass lesion, and centrilobular emphysematous changes over bilateral upper lobes. The causative organism on bronchoalveolar lavage (BAL) taken from the left lower lobe was identified to be MAC. The patient was admitted and started on ethambutol 275 mg, isoniazid 75 mg, rifampicin 150 mg once a day each, clarithromycin 500 mg twice a day, amikacin 750 mg once a day, pyridoxine hydrochloride 40 mg once a day, and a fixed-dose combination (FDC) of piperacillin with tazobactam 4.5 gram every six hours, with a high protein diet and inhalational steroids to correct the oxygen saturation levels.

On day two, the prothrombin time and international normalized ratio (PT/INR) were 23.5 seconds and 1.42, respectively, and the hemoglobin level was 10.4 gram/dL, for which he was started on tranexamic acid 500 mg every eight hours and ethamsylate 250 mg thrice a day. Other investigation findings were within normal limits and were not clinically significant. Alarmingly, the oxygen saturation levels were neither deteriorating nor improving, raising concerns for associated concomitant respiratory dysfunction. On day four, BAL samples were sent for histopathological examination (HPE) and cytology. On BAL HPE, solid ADC of the lung was noted, and tumor cells expressed thyroid transcription factor-1 (TTF-1) and napsin-A. BAL cytology revealed atypical epithelial cells, nuclear polymorphism, and an increased nuclear-to-cytoplasmic ratio, confirming the left lung’s malignancy. The patient was counseled for chemotherapy and from day six onwards, he was started on radiation therapy.

After achieving vital and hemodynamical stability, the patient was discharged on day 15 with advice to continue ethambutol 275 mg, isoniazid 75 mg, rifampicin 150 mg once a day each, clarithromycin 500 mg twice a day, amikacin 750 mg once a day, pyridoxine hydrochloride 40 mg once a day, and to maintain a high protein diet. For further specialty care with an oncologist, he was referred to a higher center.

## Discussion

MAC, commonly isolated from water, house dust, and soil, remains the most common NTM-associated pulmonary disease [2]. Modes of infection from MAC primarily include inhalation, ingestion, invasive procedures, or trauma [4]. The risk factors contributing to the development of MAC are smoking, alcoholism, immunosuppressed state due to acquired immunodeficiency syndrome, and underlying respiratory infection or carcinoma [2,4-7]. However, our presented case did not have a history of smoking or alcoholism. The patient was diagnosed with MAC infection associated with ADC leading to deterioration of symptoms even after initiating primary therapy. Treatment for nontuberculous mycobacterium often remains a challenge due to the increasing resistance against antimicrobials resulting in poor prognosis for such patients. Symptoms of MAC are often nonspecific and due to the limitation in microbial detection, they are often misdiagnosed as TB or other acid-fast bacilli-positive organisms [2]. Typical treatment of MAC includes a regimen of macrolide, rifampicin, and ethambutol [8]. Amikacin is added mainly in cases of cavitatory nontuberculous mycobacterium infection [8]. However, in cases of refractory MAC, a four-drug regimen, including macrolide, rifampicin, ethambutol, and amikacin, is suggested [8]. In our case, we opted for the above-mentioned four drugs along with an FDC of piperacillin and tazobactam, which provided promising effects initially. Inhalational steroids were successful in improving oxygen saturation by decreasing the inflammatory process and improving airflow.

However, patients with concomitant infection and carcinoma are always a diagnostic and therapeutic challenge. The majority of ADC patients are detected in advanced stages with a poor prognosis of a five-year survival rate of only approximately 15% [3]. Initiation of radiation therapy to curb the malignancy may compromise the immune system, which can lead to failure of treatment of infectious cause or relapse of symptoms post treatment. In our case, it was also a decisive challenge to initiate the radiation therapy and it was started after the patient’s antimicrobial therapy began to alleviate the symptoms.

Strength of the case

This case outlines a unique association of concomitant ADC and pulmonary MAC and provides a different outlook on the administration of therapy.

Limitations of the case

Short follow-up duration and no further status of the patient post discharge are limitations of this case.

## Conclusions

Concomitant MAC infection and ADC is a rare association. Conservative therapy often subsides the symptoms in patients with MAC; however, ADC association may lead to deterioration of the condition. Initiation of specific therapy for the malignancy is required for the improvement of the patient’s condition. Even if the mycobacteria is sensitive to the antimicrobial, failure of therapy can be due to underlying malignancy, which needs to be ruled out.
